# Involvement of personal and professional relations among men bothered by lower urinary tract symptoms: a population-based cross-sectional study

**DOI:** 10.1186/s12889-020-08992-z

**Published:** 2020-06-05

**Authors:** Marianne Møldrup Knudsen, Kirubakaran Balasubramaniam, Peter Fentz Haastrup, Dorte Ejg Jarbøl, Sanne Rasmussen

**Affiliations:** 1 University of Southern Denmark, Odense M, Denmark; 2 Research Unit of General Practice, Department of Public Health, University of Southern Denmark, J.B. Winsløws Vej 9A, 5000 Odense C, Denmark

**Keywords:** Lower urinary tract symptoms, Men, Primary health care, Help-seeking behaviour, Social network

## Abstract

**Background:**

Lower urinary tract symptoms (LUTS) are common among men worldwide and despite frequently of benign origin, the symptoms often influence quality of life. Most men experiencing LUTS manage their symptoms in private settings without consulting their general practitioner (GP). Therefore, the purpose of this study was to identify who in the personal and professional relations Danish men discussed their bothersome LUTS with, to analyse factors associated with discussing LUTS with personal and professional relations, and to analyse how having a social network influenced healthcare seeking.

**Methods:**

A nationwide population-based, cross-sectional survey representative of the Danish population. A total of 46,647 randomly selected men aged 20+ were invited to participate. Data were collected in 2012. The main outcome measures were odds ratios between involvement of personal and professional relations, GP contact and different characteristics (age, number of symptoms, available social network, and involvement of personal relations) among men experiencing bothersome LUTS. We used multivariate logistic regression models.

**Results:**

Overall, 22,297 men completed the questionnaire. Of those, 4885 (21.9%) had experienced at least one LUTS, 23.5% had not discussed their symptoms with either personal nor professional relations and 59.1% had not discussed their LUTS with any professional relation. The symptoms were most often discussed with personal relations, primarily the spouse/partner who was involved in more than half of the cases. Odds of consulting the GP, another doctor and other healthcare professionals were two to four-fold higher when the symptoms were discussed with a personal relation. Having an available social network was significantly associated with lower odds of consulting the GP regarding frequent urination.

**Conclusions:**

Despite the high prevalence of bothersome LUTS more than one-fifth of men did not discuss their symptoms with either personal nor professional relations, and more than half did not discuss the symptoms with any professional relations. Discussing the symptoms with personal relations was generally associated with higher odds of seeking professional help, and for frequent urination, having an available social network was associated with lower odds of consulting the GP. The results may be useful for detecting and treating men bothered by LUTS.

## Background

Lower urinary tract symptoms (LUTS) are common among men worldwide and despite often being of benign origin, they are associated with decreased quality of life [1]. The prevalence among men is estimated to 40–95% [2–5], and increases with age [2]. The large variation is presumably due to different definitions, methods and study populations.

LUTS include various urological symptoms and can be classified into three subgroups; storage, voiding and post micturition symptoms [6]. These symptoms are often perceived as a normal part of ageing which might prevent individuals from consulting their general practitioner (GP) [7, 8].

Benign prostate hyperplasia (BPH) is the most common cause of LUTS but several other conditions can cause LUTS, including weakness of the detrusor muscle, urinary tract infection, overactive bladder and prostate cancer [6]. The majority of causes are benign and thus not life-threatening but distressing with the potential of causing psychological effects and exacerbating feelings of social isolation [7].

Medical examination is essential to initiate an intervention that can possibly alleviate the symptoms. Although several treatments are effective in reducing LUTS [9], the majority of men manage their LUTS in private settings [3] and thus the symptoms presented to GPs are only the tip of the symptom iceberg [10]. This could partly be explained by the fact that not all men are bothered by these symptoms, hence it is understandable that they do not consult the GP.

A possible explanation for non-attendance to the GP is that men are unaware of the availability of medical treatment, and thus they should be encouraged to consult their physician if they have bothersome symptoms [11]. A study from 2004 has shown that advice from a person’s social network increased the likelihood of contacting the GP with health-related inquiries approximately fivefold [12]. In the study based on data from general practice, 25% of men received advice prior to an appointment with their GP. Spouse/partner was the most frequent person to give advice (50%) but was not significantly superior to other people from the social network, that was defined as family, friends, colleagues, neighbours, home helps and district nurses [12].

The decision to contact the GP is not simply based on the presence or absence of symptoms but is affected by multiple factors. Seeking help, and deciding with whom symptoms should be discussed, is thought to be based on a complicated decision-making process [13].

When analysing healthcare seeking regarding symptoms in general, we have previously found that people without an available social network overall were more likely to involve the GP compared to those with an available social network [14]. So perhaps feeling socially isolated increases the chances of individuals visiting their GP as a way to receive much-needed social connection [15, 16]. Based on this, we hypothesized that men without an available social network were more likely to involve the GP regarding their LUTS compared to those with an available social network.

The aim of this study was [1] to identify the personal and professional relations involved by Danish men bothered by LUTS, [2] to analyse factors associated with involvement of personal and professional relations and [3] to analyse how access to a social network influenced healthcare seeking with bothersome LUTS.

## Method

### Study design and population

This population-based, cross-sectional study is based on data from the Danish Symptom Cohort, a nationwide cohort comprising a random sample of 100,000 people aged 20 years or above who are representative of the adult Danish population.

All Danish citizens are registered with a unique personal identification number in the Danish Civil Registration System (CRS), which contains information on date of birth, gender etc. [17]. Invitees were randomly selected from the general population through the CRS and invited to participate in the survey by a letter explaining the purpose of the study. The letter included a unique login to a secure web page, which provided access to a comprehensive questionnaire. After 2 weeks a reminding letter was sent to non-respondents and after additional 2 weeks non-respondents were contacted by telephone and encouraged to participate. A telephone interview conducted by trained interviewers was offered to prevent the exclusion of people with no internet access. Data were collected from June to December 2012 and the methodological framework for developing and testing the questionnaire has been thoroughly described by Rasmussen et al. [18]. In this study, only male respondents were included.

### Questionnaire

#### Symptom related questions

The questionnaire contained 44 predefined symptoms and six of these were related to LUTS in men and included in the present study. Five of the six symptoms were related to storage (nocturia, frequent urination, urge incontinence, stress incontinence and incontinence without stress/urge) and one was related to voiding (difficulty in emptying the bladder). The questions regarding symptom experiences were phrased: “*Have you experienced any of the following bodily sensations, symptoms or discomforts within the past four weeks?*”. It was possible to report more than one symptom and for each reported symptom, the respondents were asked to provide additional information about who they had talked to regarding the symptom, and to what degree the symptom had been concerning and influencing their daily activities. A five-point Likert scale was used as response option for the concern and influence on daily activities: *not at all, slightly, moderate, quite a bit and extremely*. For each symptom reported the respondents were subsequently asked whether they had contacted their GP with the symptom or discomfort, in person, by phone or by e-mail. Additionally, respondents were asked which other healthcare professionals they had contacted with the options being: *another doctor (practicing specialist, out-of-hours physician or hospital physician), physiotherapist/chiropractor, home help/district nurse, pharmacy staff, alternative therapist (*e.g. *homeopath, healer, reflexologist), none and ‘other’ category*. Furthermore, they were asked which personal relations they had talked to regarding the symptom with the options being: *spouse/partner, children, parents, colleague/classmate, friend, neighbour, none and ‘other’ category*. More than one relation could be selected.

#### Generic questions on social network

To examine whether the respondents had an available social network four items were used: [1] “*How often are you in contact with friends, acquaintances or family that you do not live with? Contact indicates that you are together, talking with each other on the phone, writing to each other etc.”* with the response options being: *daily or almost daily, once or twice a week, once or several times a month, less than once a month, never, I don’t know* [2]. “*If you become ill and need help with practical things, can you count on help from others? Others means people you do not live with*” with the response options being: *yes, definitely, yes, maybe, no*. [3] “*Does it ever happen that you are alone, even if you want to be in the company of others?*” with the response options being: *yes, often, yes, once in a while, yes, but rarely, no*. [4] “*Do you have someone to talk to if you have problems or need support?*” with the response options being: *yes, often, yes, mostly, yes, sometimes, no, never or almost never*.

### Data analysis

**Fig. 1:**
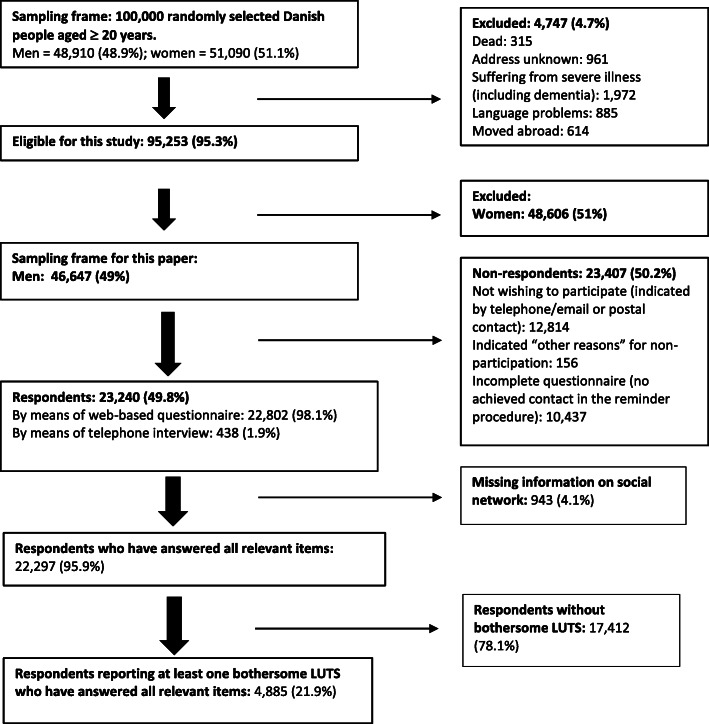
Flowchart of the study cohort (2012)

The dataset for this study comprises men with bothersome LUTS, defined as being of *moderate to extreme concern* and/or with *moderate to extreme* influence on daily activities. This means that symptoms reported as being of *no or slight* concern and/or *with no or slight influence* of daily activity were not included in the analyses. For further details of the distribution of bothersome LUTS, see Rubach et al. [19]. The study population included respondents who had answered all relevant questions (Fig. 1).

Basic descriptive analysis was used to study the involvement of each personal and professional relation when experiencing each of the following six symptoms: frequent urination, nocturia, stress incontinence, urge incontinence, incontinence without urge/stress and difficulty emptying the bladder. Subsequently, the three incontinence symptoms were merged into one symptom category named ‘incontinence’, giving a total of four LUTS (frequent urination, nocturia, incontinence and difficulty emptying the bladder). The merge was due to few observations in each symptom group, and the category included the incontinence symptoms if any of the three incontinence symptoms were reported as bothersome. The study sample was stratified into four age groups: < 40 years, 40–59 years, 60–79 years and ≥ 80 years.

Regarding the available social network, individuals were categorized as having no available social network if all the following responses were chosen: never/less than once a month being in contact with others, having no available individuals who can help, often being alone when desiring to be with others and never/almost never having a person to talk to in case of problems.

We used multivariate logistic regression models to analyse possible associations between involvement of personal and professional relations and specific characteristics (age, number of symptoms, available social network and personal relations). Crude and adjusted odd ratios were calculated. Adjustments were made for possible confounders: age, number of symptoms and available social network. For the logistic regression analyses we categorized the personal and professional relations into five groups: [1] the GP, [2] another doctor, [3] other healthcare professionals, [4] family members and [5] personal relations other than family. Other healthcare professionals comprised all professional relations selectable apart from the GP and another doctor, family members included spouse/partner, parents and children and personal relations other than family encompassed colleague/classmate, friend, neighbour and others.

All data analyses were conducted using STATA 13.1 (StataCorp) and a significance level of *p* < 0.05 was used.

## Results

Of the 48,910 randomly selected men, 46,647 were found eligible for the study and 23,240 participated, yielding a response rate of 49.8% (Fig. 1). The majority (98.1%) completed the questionnaire online. A total of 22,297 men had answered all relevant questions regarding involvement of personal and professional relations and available social network. Of those, 4885 (21.9%) men reported at least one bothersome LUTS and were included in this study (Fig. 1).

Baseline characteristics differed among respondents and non-respondents. The median age among respondents was 53 years compared to 48 years among non-respondents. More respondents were married/living together, had a high level of education, high income and were more often working compared to non-respondents [10].

Overall, the prevalence of LUTS varied from 4.1% for bothersome incontinence to 17% for bothersome nocturia (Table 1). The proportion of men who did not involve neither personal nor professional relations varied between 21.3 and 27.3% for nocturia and difficulty emptying the bladder, respectively (Table 1). When a relation was involved it was most frequently a personal relation, mainly the spouse/partner, friend and children regarding all four LUTS (Table 1). Among professional relations, the GP and another doctor were the most frequently involved regarding all bothersome LUTS. However, more than half of the men with symptoms (difficulty emptying the bladder (50.7%), frequent urination (50.5%), nocturia (60.5%), overall incontinence (54.1%)) did not seek advice from a professional relation (Table 1).

**Table 1:** Characteristics of the study sample based on the different symptoms

	Study sample		Difficulty emptying the bladder		Frequent urination		Nocturia		Stress incontinence		Urge incontinence		Incontinence without stress/urge		Over all incontinence^a^	
	n	(%)	n	(%)	n	(%)	n	%	n	%	n	%	n	%	n	%
Study sample																
Over all	22,297	(100)	1676	(7.5)	1467	(6.6)	3794	(17.0)	135	(0.6)	730	(3.3)	219	(1.0)	921	(4.1)
Age																
< 40	5303	(23.8)	166	(3.1)	201	(3.8)	350	(6.6)	11	(0.2)	45	(0.8)	23	(0.4)	71	(1.3)
40–59	8867	(39.8)	497	(5.6)	458	(5.2)	1309	(14.8)	41	(0.5)	186	(2.1)	47	(0.5)	239	(2.7)
60–79	7528	(33.8)	920	(12.2)	732	(9.7)	1928	(25.6)	66	(0.9)	444	(5.9)	126	(1.7)	539	(7.2)
> 80	599	(2.7)	93	(15.5)	76	(12.7)	207	(34.6)	17	(2.8)	55	(9.2)	23	(3.8)	72	(12.0)
Social network																
Available social network	19,650	(88.1)	1394	(83.2)	1180	(80.4)	3148	(83.0)	100	(74.1)	594	(81.4)	174	(79.5)	747	(81.1)
Professional relations^b^																
General practitioner			643	(38.4)	559	(38.1)	1095	(28.9)	62	(45.9)	251	(34.4)	82	(37.4)	320	(34.7)
Another doctor			489	(29.2)	425	(29.0)	803	(21.2)	50	(37.0)	185	(25.3)	79	(36.1)	249	(27.0)
Physioterapist/chiropractor			18	(1.1)	19	(1.3)	21	(0.6)	< 5		5	(0.7)	5	(2.3)	11	(1.2)
Home carer/nurse			17	(1.0)	13	(0.9)	26	(0.7)	< 5		8	(1.1)	9	(4.1)	18	(2.0)
Pharmacy staff			11	(0.7)	11	(0.7)	20	(0.5)	< 5		5	(0.7)	< 5		7	(0.8)
Alternative therapist			21	(1.3)	17	(1.2)	43	(1.1)	< 5		8	(1.1)	< 5		10	(1.1)
Other			92	(5.5)	76	(5.2)	222	(5.9)	7	(5.2)	37	(5.1)	18	(8.2)	50	(5.4)
None			850	(50.7)	741	(50.5)	2294	(60.5)	59	(43.7)	404	(55.3)	99	(45.2)	498	(54.1)
Personal relations^b^																
Spouse/partner			979	(58.4)	902	(61.5)	2563	(67.6)	76	(56.3)	473	(64.8)	122	(55.7)	574	(62.3)
Children			131	(7.8)	165	(11.2)	388	(10.2)	23	(17.0)	82	(11.2)	26	(11.9)	100	(10.9)
Parents			38	(2.3)	55	(3.7)	115	(3.0)	< 5		17	(2.3)	10	(4.6)	26	(2.8)
Colleague/classmate			56	(3.3)	54	(3.7)	177	(4.7)	6	(4.4)	14	(1.9)	< 5		20	(2.2)
Friend			149	(8.9)	156	(10.6)	430	(11.3)	14	(10.4)	78	(10.7)	14	(6.4)	94	(10.2)
Neighbour			28	(1.7)	37	(2.5)	86	(2.3)	6	(4.4)	14	(1.9)	< 5		18	(2.0)
Other			34	(2.0)	26	(1.8)	69	(1.8)	< 5		21	(2.9)	8	(3.7)	28	(3.0)
None			596	(35.6)	438	(29.9)	966	(25.5)	44	(32.6)	197	(27.0)	74	(33.8)	267	(29.0)
No relation																
None^c^			458	(27.3)	327	(22.3)	807	(21.3)	34	(25.2)	157	(21.5)	55	(25.1)	208	(22.6)

^a^ The three incontinence symptoms (urge incontinence, stress incontinence and incontinence without stress/urge) were merged into one group named incontinence

^b^Responses are individually based. The percentages do not total to 100 because an individual might have involved several professional and personal relations regarding the same symptom

^c^None indicates that neither personal nor professional relations were involved regarding the symptom

Involvement of personal and professional relations among men experiencing at least one bothersome LUTS stratified according to age groups is shown in Table 2. Overall, 23.5% of men experiencing at least one bothersome LUTS involved neither personal nor professional relations and 59.1% involved no professional relation at all (Table 2). Men < 40 years of age were more likely not to involve any relation (43.1%) than men > 80 years of age (11.5%). Among men < 40 years of age, 13.7% involved the GP regarding their symptom compared to 44% among men > 80 years of age (Table 2). A similar pattern for involving another doctor was found (Table 2). No involvement of any professional relation was reported more often among men < 40 years of age (77.9%) compared to men aged 80 years or above (41.9%) (Table 2).

**Table 2:** Involvement of personal and professional relations stratified according to age groups

	Study sample		< 40 years		40–59 years		60–79 years		> 80 years	
	n	(%)	n	(%)	n	(%)	n	(%)	n	(%)
Total	4885	(100)	569	(100)	1702	(100)	2380	(100)	234	(100)
Professional relations^a^										
General practitioner	1478	(30.3)	78	(13.7)	371	(21.8)	926	(38.9)	103	(44.0)
Another doctor	1089	(22.3)	58	(10.2)	268	(15.7)	688	(28.9)	75	(32.1)
Physiotherapist/chiropractor	45	(0.9)	6	(1.1)	18	(1.1)	19	(0.8)	< 5	
Home carer/nurse	43	(0.9)	< 5		8	(0.5)	23	(1.0)	10	(4.3)
Pharmacy staff	31	(0.6)	6	(1.1)	11	(0.6)	11	(0.5)	< 5	
Alternative therapist	60	(1.2)	15	(2.6)	21	(1.2)	22	(0.9)	< 5	
Other	292	(6.0)	18	(3.2)	85	(5.0)	171	(7.2)	18	(7.7)
None^b^	2888	(59.1)	443	(77.9)	1176	(69.1)	1171	(49.2)	98	(41.9)
Personal relations^a^										
Spouse/partner	3132	(64.1)	229	(40.2)	1016	(59.7)	1725	(72.5)	162	(69.2)
Children	482	(9.9)	< 5		113	(6.6)	307	(12.9)	59	(25.2)
Parents	171	(3.5)	74	(13.0)	84	(4.9)	12	(0.5)	< 5	
Colleague/classmate	233	(4.8)	28	(4.9)	115	(6.8)	88	(3.7)	< 5	
Friend	548	(11.2)	76	(13.4)	183	(10.8)	277	(11.6)	12	(5.1)
Neighbour	110	(2.3)	< 5		36	(2.1)	68	(2.9)	< 5	
Other	109	(2.2)	5	(0.9)	28	(1.6)	68	(2.9)	8	(3.4)
None^b^	1386	(28.4)	271	(47.6)	581	(34.1)	492	(20.7)	42	(17.9)
No relation										
None^c^	1148	(23.5)	245	(43.1)	515	(30.3)	361	(15.2)	27	(11.5)

The results are based on men with at least one bothersome LUTS

^a^Responses are individually based. The percentage do not total to 100 because an individual might have involved several professional and personal relations regarding the same symptom. The relations involved can vary between symptoms experienced. A relation was regarded as involved if it was involved for at least one of the symptoms experienced

^b^ None is defined as selecting ‘None’ for all the symptom categories experienced

^c^None indicates that neither personal nor professional relations were involved for any symptoms experienced

The odds of involving the GP, another doctor, other healthcare professionals, family members and other personal relations regarding each of the covariates are shown in Tables 3, 4, 5, 6. The odds of involving the GP were statistically significantly higher in the oldest age group for frequent urination, nocturia and difficulty emptying the bladder but not for incontinence. Similarly, the odds for involving another doctor was statistically significantly higher among men > 60 years of age compared to those < 40 years of age for all symptoms except for incontinence. The odds of involving a family member were higher in the oldest age groups for difficulty emptying the bladder. The same pattern was found for frequent urination although not statistically significant for the group > 80 years. (Tables 3, 4, 6).

**Table 3:** Characteristics associated with involving professional and personal relations among men with bothersome frequent urination

	Involving the GP		Involving another doctor		Involving other healthcare professionals^a^		Involving family members		Involving personal relations other than family^b^	
	Crude OR	Adj. OR (95% CI)	Crude OR	Adj. OR (95% CI)	Crude OR	Adj. OR (95% CI)	Crude OR	Adj. OR (95% CI)	Crude OR	Adj. OR (95% CI)
Age										
< 40	1 (Ref)	1 (Ref)	1 (Ref)	1 (Ref)	1 (Ref)	1 (Ref)	1 (Ref)	1 (Ref)	1 (Ref)	1 (Ref)
40–59	**1.81(1.21–2.72)**	**1.55(1.02–2.36)**	**1.88(1.20–2.97)**	1.60(1.00–2.57)	0.92(0.51–1.65)	0.79(0.44–1.44)	**1.73(1.24–2.42)**	**2.25(1.07–4.76)**	0.96(0.60–1.52)	0.73(0.44–1.20)
60–79	**3.78(2.58–5.54)**	**2.76(1.84–4.13)**	**3.46(2.26–5.31)**	**2.43(1.55–3.82)**	0.99(0.57–1.71)	0.76(0.43–1.35)	**3.29(2.38–4.54)**	**2.67(1.32–5.43)**	0.99(0.64–1.53)	0.59(0.37–0.96)
> 80	**5.30(2.99–9.40)**	**3.47(1.90–6.36)**	**3.40(1.84–6.31)**	**2.06(1.07–3.97)**	0.72(0.26–2.00)	0.50(0.18–1.45)	**4.35(2.35–8.07)**	3.88(0.83–18.17)	0.83(0.39–1.79)	0.44(0.19–0.99)
Number of symptoms										
1	1 (Ref)	1 (Ref)	1 (Ref)	1 (Ref)	1 (Ref)	1 (Ref)	1 (Ref)	1 (Ref)	1 (Ref)	1 (Ref)
2	1.16(0.84–1.60)	0.92(0.65–1.29)	1.05(0.74–1.49)	0.85(0.58–1.23)	1.21(0.69–2.13)	1.22(0.69–2.16)	**1.40(1.04–1.88)**	1.32(0.65–2.66)	0.84(0.55–1.27)	0.78(0.49–1.22)
3	**1.96(1.40–2.73)**	1.24(0.87–1.78)	**1.80(1.26–2.58)**	1.16(0.79–1.71)	1.36(0.76–2.45)	1.25(0.68–2.31)	**2.70(1.94–3.76)**	1.53(0.70–3.31)	1.15(0.75–1.77)	0.90(0.56–1.44)
≥ 4	**3.54(2.41–5.21)**	**2.14(1.42–3.23)**	**3.16(2.12–4.71)**	**2.00(1.30–3.08)**	1.59(0.83–3.05)	1.44(0.73–2.84)	**3.25(2.16–4.89)**	1.89(0.72–4.97)	1.52(0.94–2.46)	1.17(0.69–1.98)
Available social network										
No	1 (Ref)	1 (Ref)	1 (Ref)	1 (Ref)	1 (Ref)	1 (Ref)	1 (Ref)	1 (Ref)	1 (Ref)	1 (Ref)
Yes	0.87(0.67–1.13)	**0.74(0.55–0.98)**	1.00(0.75–1.33)	0.83(0.61–1.13)	0.72(0.47–1.11)	**0.64(0.41–0.99)**	**2.06(1.59–2.68)**	**2.00(1.10–3.67)**	0.91(0.64–1.30)	**0.68(0.46–0.99)**
Involvement of personal relations										
No	1 (Ref)	1 (Ref)	1 (Ref)	1 (Ref)	1 (Ref)	1 (Ref)	–	–	–	–
Yes	**3.55(2.72–4.63)**	**3.04(2.30–4.03)**	**5.11(3.66–7.14)**	**4.45(3.15–6.27)**	**2.40(1.47–3.92)**	**2.58(1.55–4.27)**	–	–	–	–

Results in bold is considered statistically significant

^a^Other health care professionals encompass all professional relations apart from the general practitioner and another doctor

^b^Personal relations other than family encompass colleague/classmate, friend, neighbor and other

Adjustments were made for possible confounders: age, number of symptoms, available social network

**Table 4:** Characteristics associated with involving professional and personal relations among men with bothersome nocturia

	Involving the GP		Involving another doctor		Involving other healthcare professionals^a^		Involving family members		Involving personal relations other than family^b^	
	Crude OR	Adj. OR (95% CI)	Crude OR	Adj. OR (95% CI)	Crude OR	Adj. OR (95% CI)	Crude OR	Adj. OR (95% CI)	Crude OR	Adj. OR (95% CI)
Age										
< 40	1 (Ref)	1 (Ref)	1 (Ref)	1 (Ref)	1 (Ref)	1 (Ref)	1 (Ref)	1 (Ref)	1 (Ref)	1 (Ref)
40–59	**1.96(1.36–2.82)**	**1.74(1.20–2.53)**	**1.63(1.09–2.43)**	1.39(0.92–2.10)	1.21(0.75–1.95)	1.08(0.67–1.75)	**1.66(1.31–2.10)**	1.55(0.86–2.80)	1.19(0.86–1.66)	0.94(0.66–1.33)
60–79	**4.84(3.41–6.86)**	**3.49(2.43–4.99)**	**3.94(2.69–5.78)**	**2.67(1.80–3.97)**	1.41(0.89–2.24)	1.09(0.68–1.74)	**3.01(2.38–3.80)**	1.49(0.85–2.62)	1.15(0.83–1.58)	0.70(0.49–0.98)
> 80	**6.32(4.09–9.75)**	**4.15(2.64–6.52)**	**4.10(2.55–6.61)**	**2.44(1.49–4.01)**	**1.86(1.01–3.44)**	1.38(0.74–2.58)	**3.59(2.41–5.34)**	2.04(0.81–5.13)	0.66(0.39–1.14)	0.37(0.21–0.65)
Number of symptoms										
1	1 (Ref)	1 (Ref)	1 (Ref)	1 (Ref)	1 (Ref)	1 (Ref)	1 (Ref)	1 (Ref)	1 (Ref)	1 (Ref)
2	**2.37(2.01–2.80)**	**2.08(1.75–2.48)**	**2.09(1.73–2.52)**	**1.81(1.49–2.20)**	**1.36(1.04–1.78)**	1.27(0.97–1.67)	**1.47(1.25–1.73)**	1.17(0.80–1.71)	**1.25(1.02–1.52)**	1.17(0.95–1.45)
3	**3.41(2.76–4.22)**	**2.70(2.16–3.36)**	**3.10(2.47–3.90)**	**2.44(1.92–3.10)**	**1.74(1.25–2.43)**	**1.54(1.09–2.16)**	**2.05(1.61–2.60)**	1.28(0.76–2.15)	**1.50(1.16–1.94)**	1.31(1.00–1.72)
≥ 4	**4.94(3.69–6.62)**	**3.78(2.79–5.13)**	**5.70(4.23–7.68)**	**4.49(3.29–6.14)**	1.60(0.99–2.57)	1.37(0.85–2.22)	**1.96(1.39–2.76)**	0.90(0.48–1.70)	**1.69(1.19–2.41)**	**1.51(1.05–2.18)**
Available social network										
No	1 (Ref)	1 (Ref)	1 (Ref)	1 (Ref)	1 (Ref)	1 (Ref)	1 (Ref)	1 (Ref)	1 (Ref)	1 (Ref)
Yes	1.00(0.83–1.21)	0.84(0.68–1.03)	1.02(0.83–1.26)	0.83(0.66–1.04)	0.95(0.70–1.28)	0.83(0.61–1.13)	**2.39(2.01–2.85)**	**2.06(1.40–3.03)**	1.08(0.86–1.37)	0.80(0.62–1.03)
Involvement of personal relations										
No	1 (Ref)	1 (Ref)	1 (Ref)	1 (Ref)	1 (Ref)	1 (Ref)	–	–	–	–
Yes	**3.79(3.08–4.67)**	**3.12(2.51–3.88)**	**5.60(4.24–7.40)**	**4.72(3.55–6.30)**	**2.77(1.94–3.95)**	**2.68(1.86–3.86)**	–	–	–	–

Results in bold is considered statistically significant

^a^Other health care professionals encompass all professional relations apart from the general practitioner and another doctor

^b^Personal relations other than family encompass colleague/classmate, friend, neighbor and other

Adjustments were made for possible confounders: age, number of symptoms, available social network

**Table 5:** Characteristics associated with involving professional and personal relations among men with bothersome incontinence^*^

	Involving the GP		Involving another doctor		Involving other healthcare professionals^a^		Involving family members		Involving personal relations other than family^b^	
	Crude OR	Adj. OR (95% CI)	Crude OR	Adj. OR (95% CI)	Crude OR	Adj. OR (95% CI)	Crude OR	Adj. OR (95% CI)	Crude OR	Adj. OR (95% CI)
Age										
< 40	1 (Ref)	1 (Ref)	1 (Ref)	1 (Ref)	1 (Ref)	1 (Ref)	**1 (Ref)**	1 (Ref)	1 (Ref)	1 (Ref)
40–59	1.23(0.66–2.30)	0.93(0.48–1.79)	1.19(0.62–2.30)	0.89(0.44–1.78)	2.89(0.85–9.81)	2.57(0.75–8.81)	**1.82(1.06–3.10)**	2.10(0.61–7.28)	1.26(0.58–2.76)	0.84(0.36–1.97)
60–79	**2.26(1.26–4.05)**	1.48(0.80–2.74)	1.67(0.91–3.09)	1.04(0.54–1.99)	2.11(0.64–6.99)	1.76(0.53–5.90)	**3.02(1.83–4.99)**	3.01(0.94–9.72)	1.15(0.55–2.41)	0.63(0.28–1.41)
> 80	2.06(0.99–4.29)	1.04(0.48–2.27)	2.04(0.95–4.37)	1.02(0.45–2.29)	3.24(0.84–12.50)	2.53(0.64–10.05)	**5.53(2.58–11.84)**	**9.98(1.02–97.71)**	0.51(0.16–1.62)	0.22(0.06–0.74)
Number of symptoms										
1	1 (Ref)	1 (Ref)	1 (Ref)	1 (Ref)	1 (Ref)	1 (Ref)	1 (Ref)	1 (Ref)	1 (Ref)	1 (Ref)
2	1.42(0.92–2.20)	1.37(0.87–2.16)	1.27(0.79–2.06)	1.21(0.74–1.98)	0.71(0.36–1.38)	0.66(0.34–1.30)	1.21(0.82–1.78)	0.90(0.33–2.46)	0.69(0.39–1.23)	0.63(0.35–1.16)
3	**2.14(1.40–3.28)**	**1.96(1.26–3.05)**	**1.93(1.22–3.05)**	**1.82(1.13–2.93)**	0.94(0.50–1.75)	0.83(0.44–1.58)	1.29(0.88–1.91)	0.91(0.33–2.51)	1.08(0.64–1.83)	0.98(0.56–1.73)
≥ 4	**3.64(2.37–5.57)**	**3.29(2.10–5.14)**	**3.29(2.09–5.18)**	**3.06(1.90–4.91)**	1.00(0.53–1.89)	0.85(0.44–1.62)	**1.66(1.10–2.50)**	1.34(0.44–4.06)	1.18(0.69–2.01)	1.06(0.60–1.88)
Available social network										
No	1 (Ref)	1 (Ref)	1 (Ref)	1 (Ref)	1 (Ref)	1 (Ref)	1 (Ref)	1 (Ref)	1 (Ref)	1 (Ref)
Yes	0.84(0.60–1.19)	0.73(0.50–1.06)	1.12(0.77–1.63)	1.01(0.67–1.52)	0.64(0.38–1.07)	0.57(0.34–0.98)	**2.46(1.76–3.44)**	**4.07(1.92–8.61)**	0.72(0.46–1.13)	**0.49(0.30–0.80)**
Involvement of personal relations										
No	1 (Ref)	1 (Ref)	1 (Ref)	1 (Ref)	1 (Ref)	1 (Ref)	–	–	–	–
Yes	**3.99(2.77–5.74)**	**3.96(2.70–5.80)**	**4.83(3.12–7.46)**	**4.63(2.96–7.25)**	**2.21(1.22–4.00)**	**2.37(1.29–4.36)**	–	–	–	–

Results in bold is considered statistically significant

^a^Other health care professionals encompass all professional relations apart from the general practitioner and another doctor

^b^Personal relations other than family encompass colleague/classmate, friend, neighbor and other

Adjustments were made for possible confounders: age, number of symptoms, available social network

**Table 6:** Characteristics associated with involving professional and personal relations among men with bothersome difficulty emptying the bladder

	Involving the GP		Involving another doctor		Involving other healthcare professionals^a^		Involving family members		Involving personal relations other than family^b^	
	Crude OR	Adj. OR (95% CI)	Crude OR	Adj. OR (95% CI)	Crude OR	Adj. OR (95% CI)	Crude OR	Adj. OR (95% CI)	Crude OR	Adj. OR (95% CI)
Age										
< 40	1 (Ref)	1 (Ref)	1 (Ref)	1 (Ref)	1 (Ref)	1 (Ref)	1 (Ref)	1 (Ref)	1 (Ref)	1 (Ref)
40–59	**2.37(1.50–3.76)**	**1.84(1.14–2.98)**	**1.97(1.20–3.23)**	1.48(0.88–2.49)	0.92(0.49–1.75)	0.74(0.38–1.43)	**2.35(1.62–3.40)**	**2.74(1.14–6.57)**	1.10(0.64–1.88)	0.61(0.33–1.11)
60–79	**4.54(2.93–7.04)**	**2.89(1.82–4.60)**	**3.38(2.11–5.39)**	**2.07(1.26–3.40)**	1.02(0.56–1.85)	0.72(0.38–1.35)	**4.60(3.23–6.55)**	**3.70(1.62–8.47)**	1.18(0.71–1.95)	0.47(0.26–0.83)
> 80	**4.84(2.70–8.67)**	**2.62(1.41–4.86)**	**4.73(2.57–8.69)**	**2.53(1.32–4.83)**	1.46(0.63–3.35)	0.93(0.39–2.22)	**6.31(3.56–11.19)**	**4.16(1.04–16.65)**	0.78(0.34–1.80)	0.26(0.11–0.63)
Number of symptoms										
1	1 (Ref)	1 (Ref)	1 (Ref)	1 (Ref)	1 (Ref)	1 (Ref)	1 (Ref)	1 (Ref)	1 (Ref)	1 (Ref)
2	**1.61(1.22–2.13)**	1.17(0.87–1.59)	**1.47(1.09–2.00)**	1.08(0.78–1.50)	0.94(0.59–1.50)	0.83(0.51–1.34)	1.77(1.37–2.28)	0.78(0.37–1.66)	1.52(1.01–2.30)	1.32(0.84–2.06)
3	**2.17(1.61–2.92)**	**1.49(1.08–2.05)**	**1.84(1.34–2.54)**	1.26(0.90–1.78)	1.03(0.62–1.69)	0.84(0.50–1.41)	2.26(1.70–3.01)	1.04(0.46–2.38)	1.52(0.97–2.36)	1.18(0.74–1.90)
≥ 4	**3.11(2.18–4.44)**	**1.95(1.32–2.86)**	**2.82(1.94–4.09)**	**1.78(1.19–2.66)**	1.37(0.78–2.42)	1.05(0.58–1.91)	2.97(2.06–4.28)	1.34(0.47–3.87)	2.16(1.32–3.54)	1.67(0.98–2.86)
Available social network										
No	1 (Ref)	1 (Ref)	1 (Ref)	1 (Ref)	1 (Ref)	1 (Ref)	1 (Ref)	1 (Ref)	1 (Ref)	1 (Ref)
Yes	1.04(0.80–1.35)	0.83(0.62–1.12)	1.05(0.79–1.39)	0.84(0.61–1.14)	0.86(0.55–1.33)	0.73(0.46–1.14)	**2.23(1.72–2.89)**	**2.60(1.38–4.87)**	1.23(0.83–1.84)	0.88(0.57–1.35)
Involvement of personal relations										
No	1 (Ref)	1 (Ref)	1 (Ref)	1 (Ref)	1 (Ref)	1 (Ref)	–	–	–	–
Yes	**4.71(3.70–6.01)**	**4.09(3.17–5.26)**	**5.51(4.13–7.35)**	**4.91(3.65–6.61)**	**3.29(2.07–5.25)**	**3.59(2.21–5.82)**	–	–	–	–

Results in bold is considered statistically significant

^a^Other health care professionals encompass all professional relations apart from the general practitioner and another doctor

^b^Personal relations other than family encompass colleague/classmate, friend, neighbor and other

Adjustments were made for possible confounders: age, number of symptoms, available social network

Report of increasing number of bothersome LUTS was statistically significantly associated with increased odds of involving the GP and another doctor for nocturia. The same tendency was found for the three other LUTS, however only significant for + 4 symptoms or 3 symptoms. (Tables 3, 4, 5, 6).

Involvement of personal relations was statistically significantly associated with a two to four-fold increased odds of involving the GP, another doctor and other healthcare professionals for all four bothersome LUTS (Tables 3, 4, 5, 6).

Having an available social network was significantly associated with lower odds of involving the GP with frequent urination (Table 3). Although not statistically significant the same tendency was seen for the remaining symptoms (Tables 4, 5, 6). For frequent urination and incontinence men with no available social network were more likely to involve a personal relation other than family (Tables 3, 5).

## Discussion

### Article summary

This population-based study comprised 22,297 randomly selected men from the general population and of those 4885 reported at least one bothersome LUTS yielding a prevalence of 21.9%. Among men experiencing bothersome LUTS, 23.5% involved neither professional nor personal relations regarding their symptom(s). Involvement of personal relations was most common and was associated with a two to four-fold increased odds of involving the GP, another doctor and other healthcare professionals. The most frequently involved professional relation was the GP followed by another doctor and the odds of involving either of these were highest among the oldest group of men. Men categorised as having no available social network had higher odds of involving the GP and other healthcare professionals regarding frequent urination. For the remaining LUTS a similar tendency was observed, however not statistically significant.

### Strengths and limitations of this study

This study was a large nationwide cross-sectional study including 22,297 male respondents. To our knowledge such a large population-based study has not previously been conducted concerning personal/professional relations and network activation among men with bothersome LUTS. However, the cross-sectional nature of the study is a limitation. Longitudinal data would allow the temporal sequence of variables to be established more strongly.The response rate of 49.8% was consistent with previous studies [3, 20]. However, it is unknown whether individuals experience symptoms are less or more inclined to participate in the study and thus an underestimation or overestimation of the prevalence cannot be eliminated.

The web-based questionnaire was not available in a paper version, which may have prevented some invitees from participating in the study, especially the elderly. The possible selection bias was minimized by offering the possibility of conducting the survey as a telephone interview to people with no internet access. The random selection through CRS was also used to eliminate the risk of selection bias.

Information on the symptom experiences and who the respondents involved regarding their symptoms were self-reported and since LUTS might be associated with shame and embarrassment [11] it is possible that an underestimation of the prevalence of LUTS is present. However, the underestimation is presumably minimized by the web-based design because of an increased perception of anonymity. The invitees were asked to recall symptom experiences within the preceding 4 weeks, and whether they had talked to a personal or professional relation regarding these symptoms at any time. The 4 weeks recall period was used to obtain statistically precise estimates while still assuming the participants could recall symptoms accurately. However, recall bias cannot completely be eliminated in questionnaire studies. Some may have forgotten to report a symptom or a relation they had involved because the symptom experience turned out to be inconsequential or simply due to memory decay. Others might have misplaced symptom experiences outside the timeframe of the study due to severity of the symptoms or because they had already involved a personal/professional relation about them. As only individuals bothered by LUTS are included we believe that this risk of recall bias is negligible [21].To obtain further information about the symptom disclosure more response options could have been presented. For instance, the use of social media or the internet might have been a frequent reason not to contact the GP among the young respondents.

This paper investigates bothersome LUTS which were defined as being of moderate to extreme concern or influence. This definition was chosen because LUTS often are of benign origin and therefore it might be unnecessary for men with no bother from their symptoms to seek medical attention. The term bothersome was a construction made in the author group based on the literature and clinical experience and is described more in detail elsewhere [19]. This construction, however, still needs further validation in the population.

To be defined as ‘bothered’ respondents could be either moderate to extremely influenced by the symptoms without being concerned or vice versa. It may have influenced the results that respondents categorised as bothered by LUTS could be moderate to extremely influenced by the symptoms without being concerned or vice versa. A diagram of the distribution of bothersome is shown in Rubach et al. [19] and it reveals that more than half of the symptoms were of both moderate/extreme influence and moderate/extreme concern. It is plausible that men being both extremely concerned and extremely influenced by LUTS are more likely to involve professional or personal relations compared to men being moderately influenced in their activities but with no or little concern. Whether influence or concern is the greater driver for involvement of others is yet to be determined.

We hypothesized that involving a personal relation may influence the decision to seek healthcare with bothersome LUTS. However, the questionnaire did not give any information about the chronological order in which the relations were involved. Therefore, we can only describe whether a relation was activated or not. Furthermore, the study did not reveal the quality or content of the contacts made and thus it is unknown whether a potential advice from the personal network is in favour of help-seeking or not. If the advice from the personal network is inadequate it may prevent the patient from contacting the GP and thus delay medical evaluation.

We intended to analyse the impact of having an available, functional network on the help-seeking process, and therefore presumed that individuals having “contact less than once a month” and “almost never having a person to talk to” had a very sparse network. In the dichotomized analyses they were therefore categorized as having no available network.

### Comparison with existing literature

Around one-third of LUTS were discussed with the GP in this study, which is lower compared to a study by Pescosolido, who found that the GP was involved in 85.4% of the “illness episodes” registered in a retrospective survey [13]. The high involvement of the GP in the study by Pescosolido was expected due to the way “illness episodes” were selected. We found that a minority of symptoms are discussed with the GP, which is in concordance with previous studies [10, 20]. Most LUTS are of benign origin and therefore treatment should primarily be targeted men with bothersome symptoms. As our survey was conducted in a gatekeeper system with free access to the GP, a high utilization of the GP was expected among our study population. However, more than half of men experiencing bothersome LUTS had not involved any professional relation. In comparison, a population-based study by Boyle et al. [22] found that 40.9–77.5% of men with bothersome incontinence involved a doctor regarding their symptom, which is higher compared to our results (34.7%). Boyle et al. [22] had a different study population of men aged 40–79 years compared to this study, where men above 20 years were included. In Boyle et al. [22] bothersome is defined based on the impact on daily life by using the BPH Impact Index whereas this present study included symptoms being either concerning and/or influencing everyday life. Further, no timeframe for experiencing the symptoms was defined in the study by Boyle et al. Norby et al. [3] found that 9.2% of men > 50 years of age with voiding problems had involved a doctor within the past 2 years, which is lower than our findings for difficulty emptying the bladder (38.4%) and may be caused by the longer timeframe, the different study populations and the severity classification. Further, they found that elderly men involved the doctor more often than the younger men [3]. This matches our study where a higher proportion of men above 80 years had involved the GP (44%) compared to men < 40 years of age (13.7%). The more frequent involvement of the GP among elderly men could possibly be caused by the fact that elderly men consult their GP more often for other reasons which provides more opportunities to present the LUTS. Likewise, the more frequent consultations could provide the elderly with a better relation to their GP and thus it could eliminate the shame and embarrassment associated with LUTS. In addition, younger men might be more likely to search the internet for advice instead of consulting the GP compared to the elderly.

Surprisingly, 77.9% of men < 40 years of age with bothersome LUTS did not involve any professional relations. Various barriers to healthcare seeking among men in general have been suggested by Yousaf et al. [23], and might be part of the explanation for this finding. Yousaf et al. found that there were several psychological and contextual factors discouraging men to seek medical help. Among these were embarrassment, anxiety and fear along with lack of knowledge about symptoms, treatments and services and viewing symptoms as minor and insignificant [23]. Furthermore, poor communication and lack of time was mentioned as important factors for initiating medical contact [23]. To what extent these factors affected our results is uncertain, but nevertheless a possible explanation for the finding.

In the present study the spouse/partner was the most frequent relation involved in general and was involved in more than half of all the cases. Friends and children followed the spouse/partner as the most common personal relation involved. These findings are supported by Roe et al., who found that spouse/partner was most frequently involved, followed by family and friends, for patients with urinary incontinence [24]. However, a lower prevalence of involving personal relations was found by Roe et al. as 47–49% had involved the spouse/partner compared to 58.4–67.6% in our study. The involvement of a friend was 25–32% in Roe et al. compared to 8.9–11.3% in our study [24]. Roe et al. included both men and women and the study were conducted in the UK, which could be the explanation for the difference in the results.

We found that odds of involving the GP, another doctor and other healthcare professionals were two to four-fold higher when a personal relation was involved. The findings are comparable to the study by Eriksson et al. [12], who found that being advised by others to seek medical attention increased the likelihood of seeking primary healthcare approximately five-fold. This might indicate that personal relations can act as a trigger of healthcare seeking.

In the present study, men without an available social network more often involved the GP about frequent urination compared to men with an available social network. Similarly, a paper with results from three studies showed that low social group connectedness was associated with a higher frequency of primary care attendance [15].

In the present study, men without a social network were more likely to involve a personal relation other than family compared to men with an available social network. The explanation for this could be that men without an available social network disclose their symptoms to a neighbour or colleague whom they cannot rely on in case of illness. Furthermore, it could indicate that family was interpreted as the available social network for most men.

## Conclusion

This study found that despite the high prevalence of bothersome LUTS more than one fifth of men experiencing LUTS involved neither personal nor professional relations, and more than half did not involve any professional relations. The involvement of relations differed with age, but overall GP and another doctor were most frequently involved among professional relations, while spouse/partner, friend and children were preferred among personal relations. Involving personal relations was associated with higher odds of involving the GP, another doctor and other healthcare professionals for all four LUTS. Among men with frequent urination, having an available social network was associated with lower odds of involving the GP compared to not having an available social network. This study delivers knowledge of symptom disclosure which is generalisable to other western cultures.Healthcare professionals could use this knowledge to detect and treat men suffering from bothersome LUTS. Likewise, this knowledge could be used in campaigns to inform the general population about the symptoms, management and treatment options available for LUTS. Such campaigns should also address spouses/partners as they were the most frequently involved personal relation and thereby, they could help encourage the men being bothered by LUTS to seek medical help. Further, the GP could use this information to take a more active approach when men consult them regarding other medical issues.

It could be relevant in future research to investigate the chronological order in which the relations are involved, the quality of the relations and the characteristics of potential barriers to involve personal and professional relations. Moreover, the use of online information sources and the social media could be further investigated.

## Abbreviations

LUTS: Lower urinary tract symptoms
GP: General practitioner
BPH: Benign prostate hyperplasia
CRS: Civil registration system
